# Respiratory morbidity in patients with spinal muscular atrophy—a changing world in the light of disease-modifying therapies

**DOI:** 10.3389/fped.2024.1366943

**Published:** 2024-03-14

**Authors:** Leen Lagae, Marijke Proesmans, Marleen Van den Hauwe, François Vermeulen, Liesbeth De Waele, Mieke Boon

**Affiliations:** ^1^ Department of Pediatrics, University Hospital Leuven, Leuven, Belgium; ^2^ Woman and Child Unit, Department of Development and Regeneration, Catholic University of Leuven, Leuven, Belgium; ^3^ Department of Rehabilitation Sciences, Research Group for Neurorehabilitation, Catholic University of Leuven, Leuven, Belgium; ^4^ Department of Development and Regeneration, Locomotor and Neurological Disorders, Catholic University of Leuven, Leuven, Belgium

**Keywords:** spinal muscular atrophy, respiratory morbidity, disease-modifying therapies, nusinersen, onasemnogene abeparvovec, risdiplam

## Abstract

Respiratory complications are common in spinal muscular atrophy (SMA) and significantly contribute to morbidity and mortality in these patients. Generalized respiratory and bulbar muscle weakness translates into diverse and complex clinical consequences necessitating strict follow-up and specialized care. The natural history of SMA has evolved drastically in recent years as a result of the introduction of novel, disease-modifying therapies. While the impact of these therapies on motor function is well described in literature, its consequence for respiratory management has not been extensively studied. In this review we aim to provide a comprehensive overview of the respiratory morbidities, their follow-up, management, and the impact of novel therapies in SMA.

## 1 Introduction

Spinal muscular atrophy (SMA) is an autosomal recessive neuromuscular disorder characterized by progressive muscle weakness and respiratory problems. The incidence is estimated to be 1 in 11 000 live births (1, 2).

Homozygous deletions or loss of function mutations in the survival motor neuron 1 (*SMN1*) gene (5q11.2–q13.3) result in SMN protein deficiency, causing degeneration of alfa-motor neurons in the anterior horn of the spinal cord (3, 4). This leads to progressive generalized muscle weakness and atrophy (3). Clinical manifestations are diverse and range from symptoms at birth with severe hypotonia, areflexia and early respiratory failure to late onset with only mild proximal muscle weakness in adulthood and minimal impact on survival (5). The genetic defect in *SMN1* causes absence of functional SMN protein in all types of SMA, but the phenotypic variability is determined by the number of copies of the *SMN2* gene. While the centromeric *SMN2* gene is nearly identical and encodes the same protein as the telomeric *SMN1* gene, only 10%–15% of its transcription results in functional SMN protein expression, due to a single nucleotide change that leads to substantial skipping of exon 7 (and thus nonfunctional SMN protein). Thus, a higher number of *SMN2* copies results in more functional SMN protein and consequently a less severe phenotype (4, 5).

**Figure 1:**
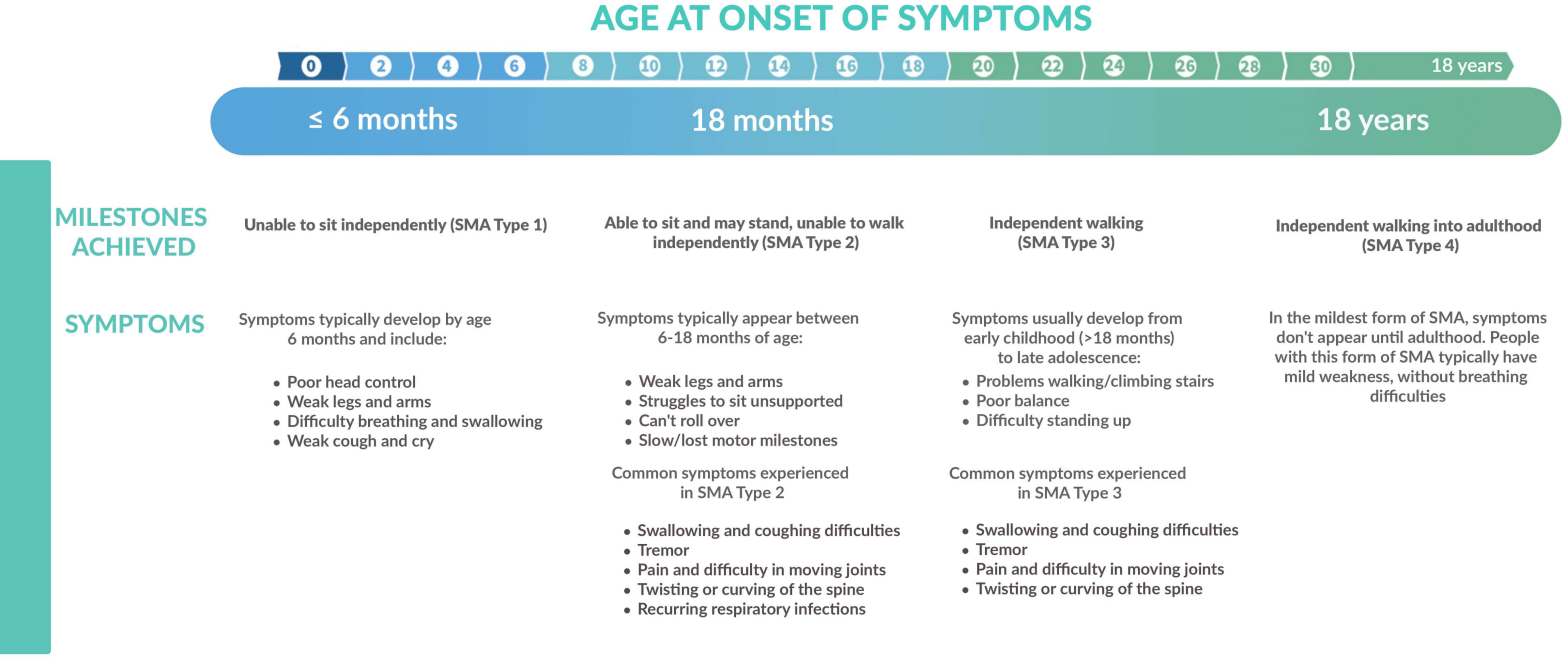
Overview of the different clinical presentations of SMA in function of age at onset and achieved milestones.

Historically, SMA was classified in different types, according to the highest achieved motor milestone and the age of onset (Figure 1) (1). SMA type I (“*non-sitters*”) is the most severe presentation in which symptoms of profound muscle weakness develop within the first 6 months of life. These children never attain the ability to sit without support as they typically present with progressive hypotonia, areflexia and limited head control. Patients with SMA type 1 suffer from a rapidly progressive respiratory failure type, historically causing dead before the age of two years (6). In SMA type 2 (“*sitters*”*)*, progressive proximal muscle weakness develops between 6 and 18 months of age and predominantly affects the lower extremities, impeding the acquisition of walking. Their respiratory phenotype is slowly progressive (6). Children with SMA type 3 (“*walkers*”*)* experience proximal weakness, mostly in the legs, starting between the age of 18 months and 18 years. They are usually spared from comorbidities and have little or no respiratory muscle weakness*.* SMA type 4 is a rare and mild phenotype, with onset of symptoms in adulthood (3, 5, 7). A very rare but severe phenotype is SMA type 0, that presents antenatally or in the neonatal period with severely decreased fetal movements. This classification in types is increasingly being abandoned because of overlap between types, its moderate predictive value on survival, respiratory and motor function, and most importantly, the changes in phenotype with the disease-modifying therapies (DMT) (8). Symptomatic patients are classified according to their motor functionality (*non-sitters*, *sitters*, *walkers*), and patients diagnosed before they have symptoms (presymptomatic patients), are classified by *SMN2* copy number.

DMT aim to increase the amount of functional SMN protein, either by targeting *SMN1* or *SMN2*. There are currently three drugs approved for SMA. Nusinersen *(Spinraza®)*, approved in 2016 by the Food and Drug administration (FDA) and in 2017 by the European Medicines Agency (EMA) as the first drug for SMA, modifies *SMN2* splicing resulting in an increased production of full-length SMN protein (9). Nusinersen is a hybridizing antisense oligonucleotide (ASO) that binds to the pre-mRNA and promotes the inclusion of exon 7. Because it does not cross the blood brain barrier (BBB), it must be administered intrathecally. After a loading period with 4 injections in two months, it is administered as a maintenance therapy every four months, lifelong (9–11). In 2019, an intravenous (IV) gene therapy, onasemnogene abeparvovec (*Zolgensma*®), was approved for use by the (FDA), followed by approval in 2020 in Europe (12). Delivery of a single IV dose of a recombinant human *SMN1* transgene by a viral vector (adeno-associated viral vector type 9, AAV9) that can cross the BBB, results in replacement of the mutated *SMN1* gene (9, 11, 13). Lastly, risdiplam *(Evrysdi®),* a small molecule that acts as a splicing enhancer, was approved in 2020 in the US and one year later by EMA (14). Similar to nusinersen, it aims to increase the amount of full length SMN protein by modification of *SMN2* splicing. As small molecules do cross the BBB, risdiplam is administered daily as a syrup.

Respiratory muscle weakness is the main cause of morbidity and mortality in patients with SMA type 1 and 2 (15, 16). Hence, an integrated understanding of the respiratory problems in SMA is essential to provide good clinical care for this population. In this review we aim to provide an overview of respiratory morbidities, their follow-up and management, and the evolution since the introduction of new, highly efficient therapies in SMA.

## 2 The multifactorial origin of respiratory problems in SMA

### 2.1 Restrictive lung disease

**Figure 2:**
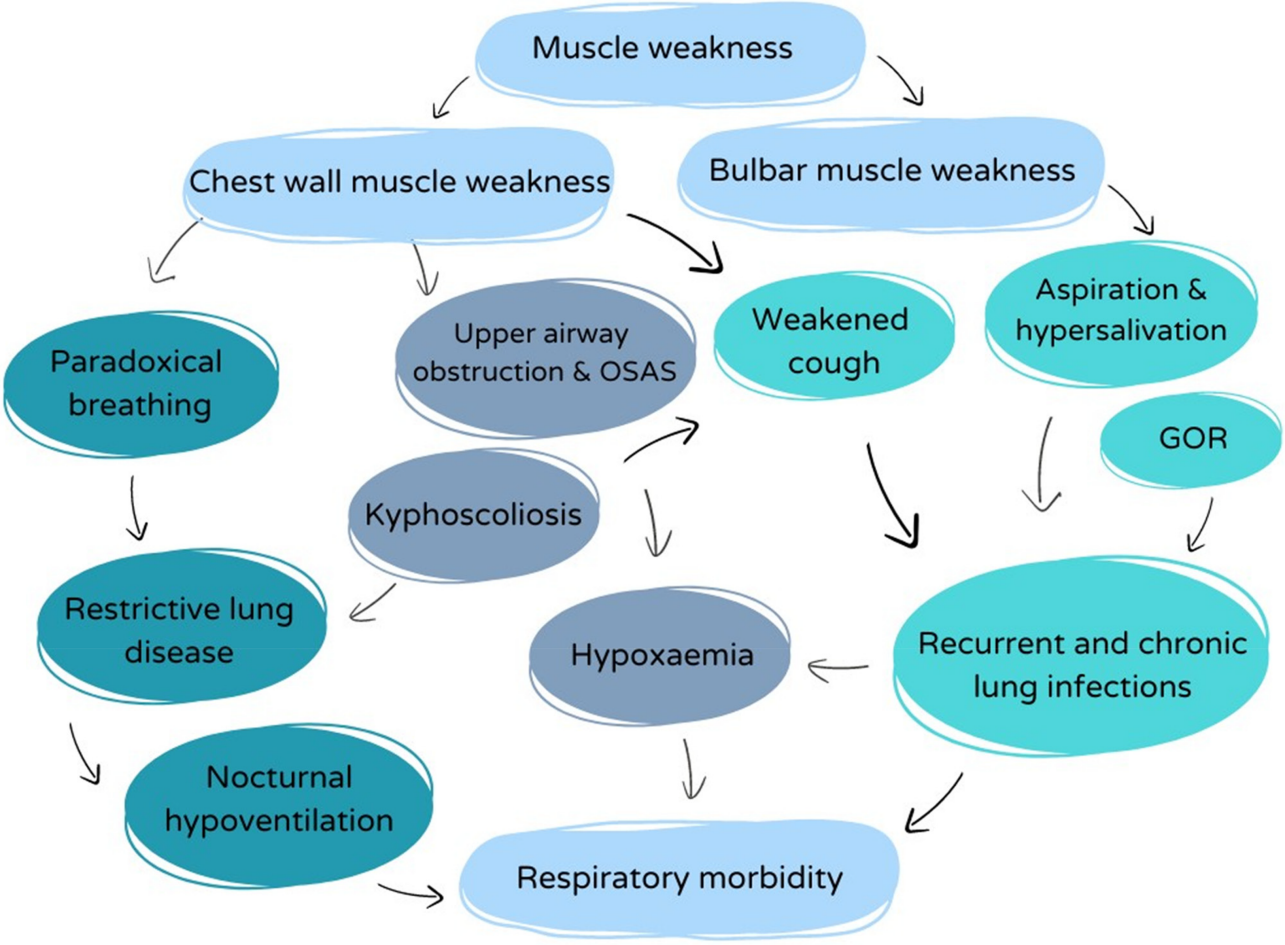
Overview of the complex interplay between respiratory factors with impact on the respiratory morbidity in SMA.

The respiratory problems in children with SMA are diverse and of multifactorial origin (Figure 2). Muscle weakness affects all muscle groups, including the inspiratory and expiratory muscles, the abdominal muscles needed for cough, as well as pharyngeal (bulbar) muscles that control the upper airway tone. However, the diaphragm function is relatively spared compared to the intercostal muscles (5). Generalized respiratory muscle weakness induces restrictive lung disease, with several clinical consequences, as illustrated below.

### 2.2 Respiratory infections

Recurrent, chronic, and severe acute respiratory infections are frequently seen in children with SMA type 1 and 2 and engender a high morbidity and mortality (17). Several factors contribute to this increased risk of lower respiratory tract infections. Most importantly, weakness of the intercostal and abdominal muscles impede cough and therefore induce inadequate clearance of airway secretions and mucus plugs from the lower airways (18, 19). Retention of bronchial secretions leads to (micro)atelectasis, subsequently increasing the risk of retro-obstructive infections (17). Because of restrictive lung disease, tidal volume ventilation is decreased, which is an additional risk factor for retention of secretions. Moreover, thoracic deformities (kyphoscoliosis, pectus excavatum—see further) reduce the thoracic elasticity and often add extra difficulty in generating a strong and effective cough.

Additionally, bulbar dysfunction may induce swallowing problems and chronic micro-aspiration leading to aspiration pneumonia. Even in patients with tube feeding because of severe swallowing dysfunction, chronic aspiration of saliva may be the cause of recurrent pneumonia.

At the time of an acute respiratory tract infection, patients with SMA will not sufficiently be capable of increasing tidal volume, but respiratory rate will increase to compensate for the higher ventilatory demand. Generally, muscle strength will further decrease at the time of an acute infection. The first sign of respiratory muscle weakness may be acute respiratory insufficiency during a severe viral lower respiratory tract infection (rhinovirus, influenza, respiratory syncytial virus, etc.), which may require invasive ventilation.

Because of weak cough, infections are slowly cleared and chronic infection, often with multi-resistant gram-negative bacteria, becomes an additional problem that can cause parenchymal damage and irreversible atelectasis and/or bronchiectasis. The most frequently isolated bacteria in SMA type 1 patients are *Pseudomonas aeruginosa* and *Staphylococcus aureus*, which suggests an inadequate clearance in between exacerbations (20). However, the clinical implication and success rate of antibiotic therapy for bacterial eradication in patients with neuromuscular diseases is unclear (17).

### 2.3 Feeding-related respiratory problems

Weakness of the oral and pharyngeal muscles leads to feeding and swallowing difficulties, and impaired airway protection against aspiration. Aspiration pneumonia often presents as acute respiratory failure. Gastro-intestinal dysmotility and gastro-esophageal reflux in particular is not infrequent in SMA, and is likely the consequence of dysfunction of the autonomic neural system. If present, gastro-esophageal reflux may increase the risk of subacute aspiration of gastric content (1, 18, 21). To avoid respiratory complications and risk of malnutrition, a gastrostomy should be considered in children with SMA type 1 and 2 at an early age (5, 17, 22). Because patients with SMA are at increased risk for catabolic status with hypoglycemia at the time of acute illness, early start of enteric feeding by gastrostomy at that time may prevent these complications. In some patients, additional anti-reflux surgery (Nissen fundoplication) is needed to decrease gastro-esophageal reflux associated respiratory problems (23–25).

### 2.4 Chest wall and spine deformities

The imbalance between the activity of the intercostal muscles and the diaphragm causes an inspiratory thoraco-abdominal asynchrony that leads to the characteristic paradoxical breathing (“belly breathing”) and a bell-shaped chest because of permanent negative intrathoracic pressure (11). Growth of the thoracic cage and lungs during infancy and childhood may therefore be suboptimal.

Almost all children with SMA types 1 and 2 develop progressive spinal deformity, and this deformity can develop early in life (3, 26). The ensuing thoracic asymmetry may increase the respiratory load by placing the already weaker breathing muscles in a mechanical disadvantage, worsening the alveolar hypoventilation (27). Additionally, kyphoscoliosis impairs cough force and, in cases of severe progression, might cause obstruction of the large airways due to torsion or external compression of the displaced spine on the trachea and/or main stem bronchi. Braces may further impede the respiration by applying extra pressure and restriction on the weak respiratory muscles and thoracic cage, and often pushing the abdominal content higher against the diaphragm. The latter may be the reason why a brace is not well tolerated in these children. The effect of spinal surgery on pulmonary function in SMA patients remains debated, as the currently available evidence is insufficient to support a beneficial effect (28). However, spinal surgery is important and aims to stabilize further progression of scoliosis and therefore further decrease in respiratory function.

### 2.5 Sleep disordered breathing (SDB)

Patients with SMA may experience nocturnal hypoventilation for several reasons. During normal sleep, overall muscle tone and central respiratory drive decrease. Therefore, the minute volume decreases, the functional residual capacity decreases and the upper airway resistance increases. Even in healthy children and adults, PaCO_2_ increases and PaO_2_ decreases during sleep. These effects are most pronounced during rapid eye movement (REM) sleep, as the muscle tone falls at its lowest in REM sleep. In SMA, inspiratory muscle weakness aggravates the decrease of tidal volume and functional residual capacity (FRC) during sleep4. As the diaphragmatic function is relatively spared, hypoventilation with CO_2_ retention will only occur in patients with severe restrictive lung disease. The presenting symptoms of nighttime hypoventilation are subtle and nonspecific: low sleep quality, disrupted sleep, morning headaches and nausea, fatigue and poor school performance.

Thoracic deformations such as scoliosis and pectus excavatum aggravate restrictive lung disease and reduce the elasticity of the thoracic cave, which is an additional factor contributing to nighttime hypoventilation. Micro-atelectasis in the context of chronic infection may as well reduce the compliance of the lung.

On top of muscle weakness, the chemoreceptors and central respiratory drive are less sensitive during sleep in SMA compared to healthy controls and thus compensation for hypoxia or CO_2_ retention is suboptimal (4, 29). Lastly, in case of chronic infection and atelectasis, ventilation/perfusion mismatch may induce impaired gas exchange.

Obstructive sleep apnea syndrome (OSAS) can be present without hypoventilation, especially in less severely affected patients. Hypotonia of the pharyngeal muscles can induce upper airway obstruction and OSAS, presenting as snoring, open mouth breathing, abnormal sleep position (for example hyperextended neck), witnessed apnea's and frequent awakenings. Symptoms include fatigue, neurobehavioral problems and even hypersomnolence during the day in severe cases. Important additional risk factors for OSAS, often forgotten, are the sleep position on the back in children with neuromuscular diseases, which increases the risk for upper airway obstruction and OSAS, and the high incidence of obesity in wheelchair bound patients (29).

### 2.6 Respiratory morbidities in the different types of SMA

The natural history of SMA type 1 is early development of respiratory failure and need for permanent ventilation support before the age of two years. Respiratory muscle weakness is prominent and restrictive lung disease is progressive. Inadequate compensation of the respiratory drive results in alveolar hypoventilation and respiratory failure (4, 30). Median age of death or permanent ventilation (defined as minimum 16 h of non-invasive ventilation per day for at least 21 consecutive days, intubation for at least 21 consecutive days, or tracheostomy) in different natural history studies is 7 to 10 months (31) (Figure 1).

Respiratory problems are also a major cause of morbidity and mortality in children with SMA type 2. Restrictive lung disease is often aggravated by comorbidities as previously described and the patients are at risk for severe and recurrent infections. Because of a slower disease progression, sleep-disordered breathing is often the first indicator of respiratory failure (1, 3).

Patients with SMA type 3 or 4 have little or no respiratory involvement (32, 33).

## 3 Pulmonary assessment

### 3.1 Respiratory follow-up

Systematic clinical follow-up of respiratory symptoms is needed in all patients with SMA 1 and 2, and for some with SMA 3 (34). During clinical assessment and from early age, attention should be paid to signs of restrictive lung disease (configuration of the thorax, kyphoscoliosis, respiratory rate and effort, paradoxical breathing, volume of the voice,…), cough force, chronic respiratory infection (abnormalities on auscultation, cough, rales or crepitations, bronchorrhea), SDB (snoring, headache, fatigue, morning nausea, bad sleep quality), aspiration (sialorrhea, cough or bronchorrhea during/after meals) and gastro-esophageal reflux (pyrosis, retrosternal pain).

Forced vital capacity (FVC) or slow vital capacity (VC), as measured by spirometry, are the most useful techniques to measure and follow restrictive lung disease and are expressed in % predicted or z-scores, in function of age, gender and height. Total lung capacity measurement is much more accurate, but sometimes harder to perform for patients. FVC evaluates both expiratory and inspiratory muscle force, as maximal expiration is also dependent of the maximal inspired volume that can be obtained. Abnormal forced expiratory volume in 1 s (FEV_1_) is a measure of airflow obstruction and can be more abnormal than FVC if airway compression is present.

In young patients or patients with severe bulbar involvement, a face mask should be used to perform spirometry. If a mouthpiece is used, it should be not too large, as many patients have reduced mobility in the temporomandibular joints. In non-ambulatory patients, calculation of z-scores is challenging. Arm span or ulnar length (less accurate) can be used to estimate height, but is often difficult to measure, especially in case of muscle contractures. Due to the limitations of performing spirometry in young children, collecting data on lung function in early-onset SMA is difficult.

Several longitudinal studies evaluated lung function decline and showed progressive worsening in patients with SMA 2 (35–37). Wijngaarde et al. studied the natural course of lung function in 170 treatment-naïve patients with SMA types 1c—4, using an extended classification with subtypes per SMA type. The natural history of FEV_1_ and FVC in early-onset SMA is characterized by progressive decrease during childhood with annual FVC decline ranging from −0,23 to −1.32% in patients with respectively SMA type 3a (age of onset 18–36 months) and SMA type 2. However, in children with SMA type 3b (age of onset >36 months) and SMA type 4, FEV_1_ and FVC remained stable.

Sniff nasal inspiratory pressure (SNIP) evaluates the inspiratory muscle force separately, and is a relatively easy manoeuver that can be performed from the age of 3–4 years. Maximal inspiratory (MIP) and expiratory pressure (MEP) at the mouth can be measured as well, and if reduced this is associated with a higher risk for severe infections. Cough force can be estimated based on peak expiratory flow (PEF), or peak cough flow (PCF). A PCF below 160 L/min is a known risk factor for inefficient mucus clearance and thus severe respiratory infections (34, 38).

When SDB is suspected based on symptoms, a polysomnography is indicated. It consists of a non-invasive but complete evaluation of sleep quality, respiratory effort, nasal and oral airflow and oxygen saturation, and should always be combined with percutaneous monitoring of CO_2_ in patients with neuromuscular disease. Because symptoms of SDB are vague and have a low diagnostic yield, polysomnography is recommended in all patients with severe respiratory muscle weakness, or other signs of respiratory insufficiency, for example recurrent respiratory infections. In general, polysomnography is recommended for patients with a neuromuscular disorder with an FVC below 60% (34, 39).

Chest radiography provides information on acute or chronic infections, and if mechanical compression of the airways in the context of a spinal deformity is suspected, a chest CT scan might be needed.

Aspiration risk can be evaluated clinically by a speech therapist, and if there is a high suspicion of aspiration, a video fluoroscopy can be performed to objectify swallowing function.

### 3.2 Pre-operative evaluation

Patients with SMA should undergo a complete respiratory evaluation by an anesthesiologist and pulmonologist before major anesthesia, in combination with a cardiologic and nutritional evaluation.

Bulbar dysfunction, together with respiratory disease and anatomical issues like limited mobility of the cervical spine or reduced mouth opening due to temporomandibular joint contractures, can cause difficulties with intubation*.* A sleep study can be considered in the pre-operative work-up to screen for subclinical nighttime hypoventilation and OSAS. Weaning from ventilation is often impaired in patients with severe restrictive lung disease. In these cases, pre-operative initiation and training of the use of non-invasive ventilation (NIV) must be considered as NIV can be used as a bridge to spontaneous breathing after extubation (40).

Higher intubation risk and ethical dilemmas that may arise in acute settings should be avoided when planning elective surgery by creating a peri-operative plan, after consulting the parents and caregivers (41).

## 4 Symptomatic treatment

### 4.1 Acute infections

Adequate respiratory physiotherapy to support mobilization of mucus and cough clearance, sufficiently aggressive treatment of acute infections with antibiotics and evaluation of the use of mucolytics are the most important therapeutical options to resolve acute infections (8, 34).

Airway physiotherapy consists of a combination of manual drainage techniques, assisted autogenic drainage, and devices that apply a positive expiratory pressure (for example PEP mask). To improve cough efficacy, devices such as mechanical insufflation-exsufflation (MI-E) techniques (cough assist) may be lifesaving. They are often combined with intrapulmonary percussive ventilation (IPV) techniques. While IPV applies oscillating positive pressure, in order to mobilize peripheral secretions to the more central airways, MI-E applies a forced positive pressure, followed by a forced negative pressure, mimicking a normal cough. It can be applied in young children and even infants, but cooperation improves tolerability. Lung volume recruitment or air stacking is a technique aimed to insufflate supplemental air volume into the lungs by using a resuscitation bag mounted on a one-way positive pressure valve and an oronasal mask. The cooperative patient is asked not to expire between several inspirations. In this way the FVC is increased and expiratory flow during cough improves. A combination of IPV, cough assist and chest compressions often is the most effective way to improve mucus clearance. In some cases, simple aspiration in the mouth is enough to remove secretions that are brought proximal after cough (34, 39, 42).

Antibiotics should be started early at the time of an acute respiratory infection, and if possible, adapted in function of previous and current respiratory cultures. Although viral infections are often the initial infectious agent, patients with poor cough are at high risk for bacterial surinfection (20). The use of mucolytics is controversial, as those with very weak cough will not be able to expectorate more liquefied, but higher volumes of sputum. Therefore, its use should be decided on an individual basis and in function of the viscosity of the secretions. If oxygen therapy is started, CO2 should be monitored as hypoxia can be the consequence of hypoventilation (34).

### 4.2 Prevention of respiratory infections

Prevention of respiratory infections is one of the main treatment goals in patients with SMA. Airway clearance therapy should be applied as preventive therapy and intensified at the time of acute infections. Protection against respiratory infections includes pneumococcal and yearly influenza vaccination, and if reimbursed, prevention of severe RSV infection with monoclonal antibodies might be beneficial as well until the age of 2 years (34, 43).

If hypersalivation in combination with severe bulbar dysfunction is apparent, treatment with anticholinergic drugs (oral glycopyrronium bromide or transdermal scopolamine) can reduce the production of saliva. A more definitive solution in patients that are exclusively fed by tube feeding, is infiltration of the salivary glands with botulinum toxin or ligation of the secretory ducts, but there is little scientific evidence to support this approach (44).

Optimization of nutritional status is important: low body mass index (BMI) and poor nutritional status can increase the perioperative risk of invasive surgery and increase the risk for severe respiratory infections, but on the other side of the spectrum, obesity can introduce SDB and even OSAS.

In patients with recurrent lower respiratory tract infections, maintenance antibiotic treatment, for example with azithromycin, can be considered (34). This has in our experience a positive effect in many patients, again without sufficient scientific evidence to support this strategy. Comorbidities like asthma and allergy are frequent, but might be difficult to diagnose because the symptoms overlap with hypotonia-induced respiratory symptoms.

### 4.3 Ventilation strategies in SMA

Positive pressure ventilation supports the inspiratory muscle force and improves ventilation dynamics. In patients with SMA type 1, respiratory failure occurred historically before the age of 2 years without respiratory intervention and the only options were tracheostomy placement with permanent invasive ventilation, or palliation (45). For patients with milder disease not in need of permanent ventilation, non-invasive ventilation (NIV) is now accepted as the ﬁrst line treatment, mostly only necessary during sleep. NIV is applied by a nasal or oronasal mask and has several benefits: efficient control of ventilatory abnormalities and respiratory symptom relief, prolonging survival and facilitating care in a home setting (3, 45). Spontaneous and triggered ventilation programs are used. Positive inspiratory (IPAP) and expiratory pressures (EPAP) are set to increase the tidal volume during quiet breathing, and consequently improve gas exchange and reduce respiratory rate. In patients with upper airway obstruction, EPAP should be set high enough. In addition, positive pressure ventilation improves the chest shape in children with SMA type 1, when using sufficiently large pressure support (difference between IPAP and EPAP) (46, 47). Consequently, besides control of gas exchange, NIV is also used to prevent the further evolution of restrictive lung disease by chest deformity and correct thoraco-abdominal asynchrony. Pressures should thus be set to reach complete control of ventilatory parameters and reduce work of breathing in the absence of mask leaks. If there is still respiratory instability with full-time NIV, tracheostomy tube placement and invasive ventilation may be unavoidable. As the tidal volume is increased with NIV, it can also be helpful to improve cough clearance and prevent infections in patients with recurrent infections. If feasible, NIV can also be used for the treatment of acute respiratory insufficiency at the time of infections in SMA type 1 and 2.

Limitations of non-invasive mask ventilation are the appearance of leaks, pressure wounds in the face, aerophagia and if started at a young age, reduced maxillary growth and therefore midface hypoplasia (48). These potential side effects should be strictly followed and can most often be resolved by changing or alternating different types of interfaces.

Adenotonsillectomy can be considered in young patients (typically around the age of 2–8 years) with SMA and OSAS, as it is the first step in the treatment of OSAS, even in patients with comorbidities, if adenotonsillar hypertrophy is clinically present (49). If this results in insufficient improvement, non-invasive continuous positive pressure ventilation (CPAP) or bi-level NIV must be applied.

### 4.4 Multidisciplinary care

Patients with SMA should be followed by a multidisciplinary team, consisting of a (pediatric) neurologist, a (pediatric) pulmonologist, a physiotherapist with respiratory and motor physiotherapy experience, a speech therapist, a (pediatric) gastro-enterologist, an ear-nose-throat specialist, a sleep specialist, an orthopedic surgeon, an anesthesiologist with experience in neuromuscular patients, an intensive care specialist, a specialized nurse, psychologist and social worker, all coordinated in a neuromuscular team. Proactive introduction of respiratory support and physiotherapy should be pursued in order to reduce morbidity and prevent secondary, sometimes irreversible respiratory damage.

## 5 Impact of disease-modifying therapies on respiratory outcomes

Several studies with real-world data, in addition to clinical trials, demonstrated efficacy of the new therapies on motor outcomes. However, the impact on respiratory function is still sparsely described (50). Here, we provide an overview of what is known on the impact of DMT on respiratory outcomes in SMA.

### 5.1 Nusinersen (Spinraza®)

The two pivotal randomized controlled clinical trials with nusinersen (ENDEAR trial for SMA type 1, CHERISH trial for SMA type 2 and 3) documented great improvements in motor function and survival (9). In the ENDEAR trial, children in the nusinersen group had a higher likelihood of event-free (defined as being alive without permanent assisted ventilation) and overall survival. Clinically significant benefits were observed in individuals in whom treatment was started early in life (10, 51, 51). The findings focused primarily on survival, the need for ventilation, and evolution in motor milestones. More detailed respiratory outcomes were not described. In the phase II NURTURE trial, the research group reported data on infants diagnosed (by newborn screening or presence of a sibling with SMA) and treated before onset of symptoms, and emphasized the importance of starting early with therapy (51). Of the 25 patients included, no patient required permanent ventilation and only four infants needed respiratory support for 7 or more consecutive days during acute illness in a median follow up period of 2.9 years (52).

Data on the impact of nusinersen on respiratory outcomes in the real-world setting are scarce and contradictory (53). A multicenter study of Farrar et al. compared the effect of nusinersen in eight newly diagnosed children with SMA type 1 vs. eight previously diagnosed and symptomatic patients. None of the patients in the newly diagnosed group required ventilatory support 5 months after the start of nusinersen, while 7 of the 8 children in the symptomatic group were started on NIV (54). These results reflect again that starting early can maximize the effect and prevent ongoing motor neuron loss. Several single-center studies also showed a delayed introduction of ventilatory support in SMA type 1 patients when treated with nusinersen (55). In a recent study of Ergenekon et al. none of the 52 patients with SMA type 1 were using NIV more than 16 h/day after 180 days of nusinersen (56). In many other single center studies, most children still required intermittent ventilatory support or even permanent ventilatory support after a wider time interval (50, 57, 58). Pechmann et al. described an increase in need of intermittent ventilatory support with age in patients with early-onset SMA, despite therapy with nusinersen. In this cohort, a lower probability to start with respiratory support after treatment with nusinersen was observed in patients with three *SMN2* copies compared to patients with two copies (59).

Stabilization of FVC after initiation of nusinersen was documented in 2 single center studies (53, 60). This stabilization was confirmed by Chako et al. with the added benefit of significantly slower rate of annual decline in the FVC z-score, compared to the retrospectively collected rate of decline in the same patients before initiation of nusinersen, especially in SMA type 2 (61).

A small French case control study assessed the respiratory muscle performance in children with SMA type 2 during a maximal sniff test. The strength of the external intercostal muscles was significantly better after six injections of nusinersen compared to historical controls (62).

Despite the stabilization in FVC and respiratory requirements, children continue to develop respiratory comorbidities including sleep disordered breathing (53), but a significant reduction in Apnea Hypopnea Index (AHI) and an improvement in oxygen nadir after introduction of nusinersen has been described (53, 61).

Data on hospitalizations due to respiratory exacerbations are diverse and inconclusive. For example, after two years of therapy, the number of hospitalizations and emergency room visits among nine children with SMA type 1 in a small Australian study decreased significantly. However, Gonski et al. reported an increase in SMA type 1 hospital admissions and no improvement in number of admissions in children with SMA type 2 and 3 following the same period (50, 53, 59). More research on this outcome is required.

### 5.2 Onasemnogene abeparvovec (Zolgensma®)

In the first open-label phase I trial (START trial), several outcomes were documented after IV treatment with onasemnogene abeparvovec in 15 symptomatic patients with SMA type 1. At 20 months of age, all patients were alive, free from permanent ventilation and achieved motor milestones that no patients in historical cohorts ever reached (63). The efficacy of onasemnogene abeparvovec was confirmed in the multicenter phase III trials (STRIVE US and STRIVE EU) with comparable results in patients younger than 6 months of age with SMA type 1 (64, 65). Even 3 years after treatment in the START trial, patients were still without permanent respiratory support and the effects on motor function were found to be sustained (13).

Real-world data regarding the efficacy of onasemnogene abeparvovec are still limited because of the only recent approval and implementation of the drug. Analyzing the effectiveness of onasemnogene abeparvovec in children who have already been treated with nusinersen presents another challenge. Published data support the drug's effectiveness in motor and respiratory outcomes (13, 66).

The advantage of newborn screening and presymptomatic diagnosis was proven in the SPRINT trial including presymptomatic infants with biallelic mutations in *SMN1* and two or three *SMN2* copies, treated with onasemnogene abeparvovec before the age of 6 weeks. The treatment significantly altered the natural course of the disease and resulted in no need for respiratory support and a nearly normal motor development in most patients at 24 months of age (67).

### 5.3 Risdiplam (Evrysdi®)

In trials that are currently accessible, only the need for permanent ventilation and survival with or without invasive or noninvasive respiratory support are examined as a proxy for respiratory morbidity after treatment with risdiplam.

In the FIREFISH multicenter open label trial, risdiplam was a safe and effective drug in symptomatic infants with SMA type 1. Ninety percent of the patients were still alive without permanent ventilation one year after initiation of treatment (14). The SUNFISH trial reported a significant clinical motor improvement in younger children and at least stabilization in older patients with SMA type 2 and 3 (68).

The above-mentioned trials included treatment-naïve patients while the JEWELFISH trial is investigating risdiplam in adult and pediatric patients who received prior treatment with another DMT. In an interim analysis, a two-fold increase in SMN protein blood levels was measured after 4 weeks of treatment, which is consistent with results in trials with treatment-naïve patients (69). Both the JEWELFISH trial and the RAINBOW FISH trial are still ongoing. The latter included genetically confirmed but presymptomatic infants. The early efficacy data are promising (14). We eagerly await further results and real-world data regarding therapeutic efficacy of risdiplam, an “at home” oral drug for SMA.

## 6 Discussion

In this review, we gave an overview on the respiratory morbidity in patients with SMA and the influence of new therapies on respiratory outcome.

Historically, respiratory morbidity and mortality was important in patients with SMA, especially those with SMA type 1 and 2. Respiratory infections and sleep disordered breathing are the consequence of respiratory muscle weakness and are the most important complications.

Strict follow-up of the respiratory evolution by multidisciplinary team evaluations, preventive treatment and specialized care are therefore needed. Nevertheless, there is no consensus as to the optimal respiratory outcome measures for assessing respiratory muscle weakness in young children with SMA (1, 4). Similarly, there is no agreement on the best timing for sleep studies to assess for early signs of respiratory failure in sleep (4). There are no internationally agreed clinical and/or overnight polysomnography (PSG) criteria for the most appropriate time to start NIV in children with SMA. Decisions to start or cease NIV are largely based on clinical judgement, based on PSG results and clinical factors including recurrence of respiratory infections, work of breathing, chest wall deformities and respiratory symptoms (43).

Since the introduction of DMT for SMA, outcomes are improving impressively. Respiratory morbidity improves, illustrated by longer survival (which is mainly a respiratory effect) and decrease in ventilatory dependence. However, many respiratory outcomes have not been studied in detail yet. For example, bulbar function seems to improve/stabilize less with nusinersen compared to other motor outcomes, probably because of a relatively reduced effect on high cervical innervated muscles compared to lumbar innervated muscles after lumbar injection. An ongoing clinical trial, the DEVOTE study, is currently investigating the efficacy and safety of a higher dose of nusinersen. It is important to investigate whether respiratory and bulbar outcomes will improve through a better bioavailability in the bulbar or higher cervical motor nuclei. It must be noted that most of the clinical trials and real-world studies were performed during the COVID pandemic. It is not unthinkable that hygienic measures indirectly improved the respiratory outcomes of patients included in these studies.

As illustrated, the effect size of DMT is highly dependent on the motor and respiratory status at the time of initiation of treatment. For those who are treated when symptoms are already established, the expectations should be moderated to stabilization or maybe some improvement. In patients with SMA type 1 diagnosed with symptoms, the phenotype will most likely evolve to a SMA type 2-like (*sitters*) phenotype, the so called “treated SMA” phenotype. These patients still often bear the burden of restrictive lung disease, with risk of frequent infections and SDB. The long term evolution of this “treated SMA” phenotype is currently unclear and the within patient variability in response has been poorly studied. Therefore, an early, presymptomatic diagnosis of SMA can enable an optimal effect of DMT, and thus, newborn screening for SMA should be implied universally with high priority. Only in this way early presymptomatic start and thus irreversible motor and respiratory damage can be prevented.

Moreover, several effects of progressive muscle weakness might not be reversible anymore at the start of DMT in older children, namely kyphoscoliosis, chronic infections with gram negative bacteria, and bronchiectasis or chronic atelectasis.

The changing landscape of SMA treatment brings hope, but also uncertainty for parents ànd caregivers. There are no data to predict treatment response to DMT, and, it might be difficult for parents of a symptomatic SMA type 1 patient to deal with this uncertainty. The therapeutic options including the expected risks and outcome must be discussed in detail, and the option of palliative care for severely affected children should still be offered.

To have a better view on changes in respiratory outcome after introduction of DMT, more specific and more detailed outcome parameters should be studied: the burden and frequency of respiratory infections, the need for hospitalizations, change in need for respiratory physiotherapy and antibiotic treatment, lung function decline, cough efficiency, bulbar function, reduction of paradoxical breathing, improvement of SDB,… Only long term follow-up studies will be able to evaluate disease progress in detail.

All DMT are very expensive and therefore reimbursement is not universal. This raises economic and ethical discussions about the maximal affordable price to improve survival and quality of life, which is currently a point of discussion for several inherited diseases.

In conclusion, the introduction of DMT has changed the field of SMA a lot in recent years, posing new challenges for adequate respiratory management of the “treated SMA” patient. Many questions remain unanswered for children that will be treated presymptomatically or shortly after onset of symptoms. Will we still have to start ventilation for optimal thoracic cage growth? Will the risk of aspiration and infections sufficiently improve? Based on what criteria can NIV be stopped? Will the treatment effect of DMT be sustained over long term (>10 years)? Could there be a role for treatment with onasemnogene abeparvovec in adolescents or adults with SMA type 2/3 with progressive respiratory disease? New clinical trials, close clinical multidisciplinary follow-up and real life follow-up studies will remain important in order to answer these questions.
